# Optimized Cas‐SF01 gene‐editing toolbox shortens flowering timing in commercial maize inbred JING724

**DOI:** 10.1111/jipb.70264

**Published:** 2026-04-22

**Authors:** Mengyuan Liu, Yao Wang, Lu Zhang, Xiang Zhang, Xiaqing Wang, Xinxiang Liu, Yue Fu, Xiaoxu Li, Ziheng Song, Ya Liu, Ronghuan Wang, Jiuran Zhao

**Affiliations:** ^1^ Beijing Key Laboratory of Maize DNA Fingerprinting and Molecular Breeding, Maize Research Institute, Beijing Academy of Agriculture and Forestry Sciences Beijing 100097 China

**Keywords:** Cas12 family, Cas‐SF01, flower time, maize, ZmCCT

## Abstract

The gene‐editing tool Cas‐SF01 was optimized to maximize its efficiency in maize. The Cas‐SF01‐TREX2 configuration was superior in enabling high‐purity gene mutations. This toolkit enabled commercial maize to flower seven days earlier without yield loss, thereby securing harvests and accelerating crop breeding.

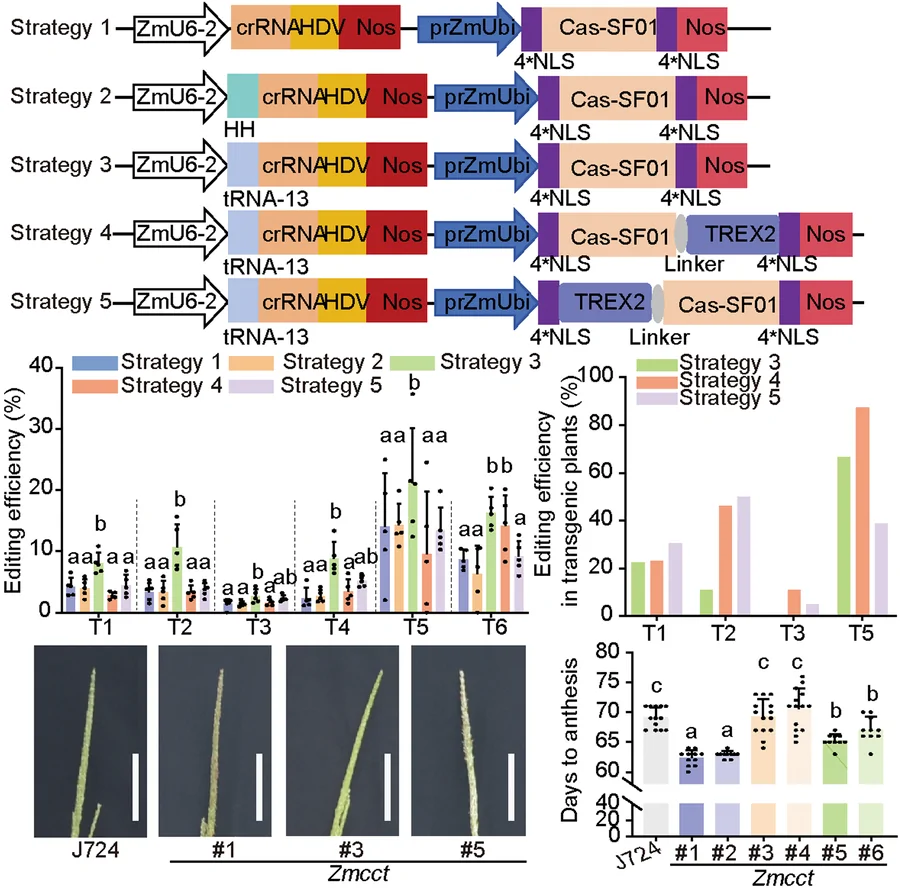

Maize (*Zea mays*) is a globally indispensable crop, serving as a major source of food, feed, and fuel. Improving quantitative traits requires new breeding strategies to overcome limited natural variation. The CRISPR/Cas12i3 system involving a Class II, type V–I CRISPR/Cas nuclease, represents a promising alternative to established genome‐editing tools. Cas12i3 is a single‐RNA‐guided nickase generating 5′‐staggered breaks at 5′–TTN–3′ PAMs (Zhang et al., 2020). Its compact nuclease (1,045 aa) and short crRNA (~43 nt) make it highly suitable for plant transformation. An engineered variant, Cas‐SF01, has been used in rice, soybean, and wheat (Duan et al., 2024; Wang et al., 2025a, 2025b), but its utility in maize remains unreported.

To establish an efficient Cas‐SF01‐mediated genome‐editing system in maize, we evaluated Cas‐SF01 activity at six endogenous targets using Strategy 1 (Figure 1A). Initial experiments with standard 23 bp spacers yielded low efficiencies of 0.21%–0.84% (Figure 1B). We therefore optimized four variables across Strategies 1–5 (Figure 1A): spacer length, temperature, crRNA arrays, and TREX2 exonuclease positioning. In maize protoplasts, 20 bp spacers increased editing efficiencies to 0.45%–1.55%, 1.7‐ to 4.89‐fold higher than 23 bp spacers (Figure 1B). Furthermore, Cas‐SF01 editing efficiencies were 3‐ to 22‐fold higher at 28°C (0.38%–18.68%) than at 22°C (Figure 1C). Evaluation of crRNA arrays showed that the tRNA–crRNA–HDV configuration (Strategy 3) yielded 8.01%–21.11% efficiency, 2‐ to 3‐fold higher than Strategies 1 and 2 (Figure 1D). Previous studies showed that most double‐strand breaks repaired by the error‐prone nonhomologous end‐joining pathway are precisely rejoined, while Three Prime Repair Exonuclease 2 (TREX2) can increase target gene mutagenesis by processing a subset of double‐strand breaks (Certo et al., 2012; Pan et al., 2025). Therefore, we fused TREX2 downstream (Strategy 4) and upstream (Strategy 5) of Cas‐SF01 via a linker peptide to ensure co‑expression of monomeric Cas‑SF01 and TREX2 (Figure 1A). In protoplasts, these fusions yielded two‐ to threefold lower efficiencies (1.60%–14.20%) than Strategy 3, although both generated longer deletions (> 20 bp) (Figure 1D, E). Conversely, in stable transgenic lines, Strategy 4 (Cas‐SF01–TREX2) markedly outperformed Strategies 3 and 5, reaching 10.81%–86.96% efficiency (Figure 1F; Table S2). Notably, Strategies 4 and 5 generated biallelic/homozygous lines (up to 55.00% and 40%, respectively) entirely absent in Strategy 3, with Strategy 4 showing minimal chimerism (one vs. seven lines; Figure 1G). For multiplex editing (3X), 3X//Strategy 4 similarly surpassed 3X//Strategy 3 by achieving higher simultaneous editing (3.70%–20.37%), elevated biallelic/homozygous rates (up to 18.18%), and zero chimerism (Figure 1H, I; Table S3). Thus, Strategy 4 establishes a robust platform for efficient, high‐purity single and multiplex maize genome editing.

**Figure 1:**
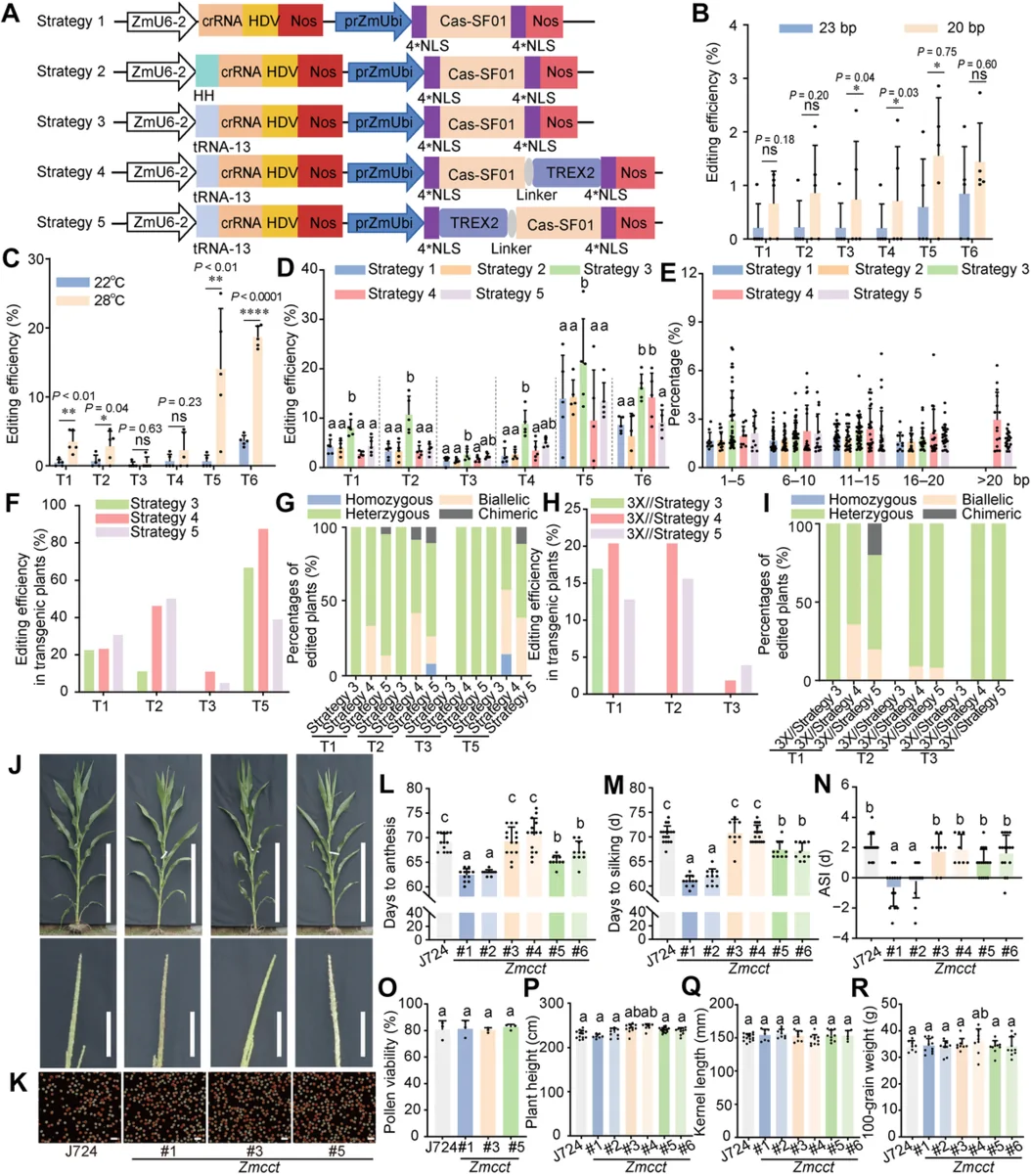
Optimization of the Cas‐SF01 gene‐editing toolbox and development of early‐flowering elite maize germplasm JING724 **(A)** Schematic diagrams of Strategies 1–5 for optimizing the Cas‐SF01 gene‐editing toolbox. **(B)** Editing efficiency of Strategy 1 with 23 bp and 20 bp spacers at six endogenous targets in the maize protoplast system. **(C)** Editing efficiency of Strategy 1 at 20°C and 28°C in the maize protoplast system. In **(B**, **C)**, statistical significance is indicated by asterisks. *P* > 0.05 means not significant (ns), and *P < 0.05, **P < 0.01, ***P < 0.001, and ***P < 0.001, Student's *t*‐test. All data represent the means ± *SD* of biological replicates. Each dot represents a biological replicate. **(D)** Editing efficiency of Strategies 1–5 at 28°C with 20 bp spacers in the maize protoplast system. **(E)** Summary of deletion pattern percentages produced by Strategies 1–5 for six targets. **(F)** Editing efficiency of single‐target Strategies 3–5 with 20 bp spacers at four endogenous targets in T_0_ transgenic plants. **(G)** Percentages of homozygous, heterozygous, biallelic, and chimeric line edited T_0_ transgenic plants generated by single‐target Strategies 3–5 with a 20 bp spacer at four endogenous targets. **(H)** Editing efficiency of transgenic plants harboring mutated alleles using three tandem tRNA–13–crRNA–HDV cassettes (3X//Strategies 3–5) at three endogenous targets. **(I)** Percentages of homozygous, heterozygous, biallelic, and chimeric edited T_0_ transgenic plants generated by 3X//Strategies 3–5 at three endogenous targets. **(J**, **K)** Whole‐plant morphology and anther dehiscence of maize tassels **(J)** at 62 d after sowing and pollen viability staining during the peak shedding period **(K)** of *Zmcct* #1, #3, #5, and JING724 in the 2024 Beijing summer. Scale bars, 1 m, 10 cm, and 200 mm, respectively. **(L**–**N)** Days to anthesis **(L)**, days to silking **(M)**, and anthesis–silking interval (ASI) **(N)** of *Zmcct* #1–#6 and JING724 in the 2024 Beijing summer. **(O)** Relative pollen viability of pollen shown in **(K)**. Around two hundred pollen grains of each genotype were assayed. **(P**–**R)** Plant height **(P)**, kernel length **(Q)**, and hundred‐grain weight **(R)** of *Zmcct* #1–#6 and JING724 measured in the 2024 Beijing summer. In **(D**, **E**, **L**–**R)**, statistical significance was evaluated by one‐way ANOVA; different letters indicate significant differences at *P* < 0.05. Each dot represents a biological replicate. All data represent the means ± *SD* of biological replicates.

We next targeted *ZmCCT* to modulate flowering time of JING724, which is the late‐flowering parental line of commercial hybrid JINGKE968, and requires accelerated flowering and a reduced anthesis‐silking interval (ASI) for reliable seed set and increased yield. *ZmCCT*, a homolog of the rice photoperiod regulator *Ghd7*, influences the flowering time (Hung et al., 2012). A CACTA‐like transposable element approximately 2 kb upstream of *ZmCCT* represses gene expression and decreases photoperiod sensitivity, thereby decreasing the number of days to flowering (Yang et al., 2013). We hypothesized that using Strategy 4 to edit *ZmCCT* will fine‐tune the flowering time of JING724. We targeted three *ZmCCT* loci using Strategy 4: exon (T1), 5′‐UTR (T2), and promoter (T3) (Figure S3A). Each target was cloned into vectors for the optimized CRISPR/Cas‐SF01 editing toolbox (Strategy 4) (Figure S2) and introduced into JING724 by *Agrobacterium tumefaciens*‐mediated transformation (Wang et al., 2026). Positive T_0_ plants were identified, and NGS identified six T_3_ homozygous mutants lacking Cas‐SF01 (Figure S3). Sanger sequencing confirmed deletions of 4–31 bp across targets (Figure S3E–G). qPCR showed that #5 and #6 (promoter mutants) had a 30%–32% reduction in *ZmCCT* expression, while expression in exon (#1/#2) and 5′‐UTR (#3/#4) mutants remained unchanged (Figure S3H). In field trials (Beijing and Sanya, 2024), whole‐plant phenotypes did not differ significantly from JING724 (Figure 1J). However, tassels of *Zmcct* #1 and #5 shed pollen earlier (Figure 1J). Statistically, mutants #1, #2, #5, and #6 flowered 3–7 d earlier (62.36–67.10 d to anthesis) than JING724 (69.08 d) (Figure 1L), with no observable changes to pollen fertility (Figure 1K, O) or plant height, kernel length, and 100‐grain weight (Figure 1P–R). Silking similarly occurred 3–9 d earlier for these mutants (Figure 1M), and ASI was notably reduced in #1 and #2 (Figure 1N). Mutants #3 and #4 (5′‐UTR) showed no obvious flowering time changes. Hence, *ZmCCT* mutations, especially in coding sequences, safely accelerated maize flowering by up to 7 d without yield penalties.

Although protoplasts are useful for primary screening, editing efficiencies differed substantially between transient and stable systems. The smaller Strategy 3 (lacking TREX2, ~700 bp shorter) likely favors transient episomal overexpression. Conversely, genomic integration in stable lines enables endogenous‐like expression, rendering the TREX2‐fused Strategies 4 and 5 superior. Thus, a two‐step validation pipeline combining both systems is critical. Furthermore, while the 20 bp spacer enhances efficiency (Figure 1B), its shorter length theoretically increases off‐target risks. However, sequencing of computationally predicted off‐target sites revealed no non‐specific mutations in *ZmCCT* mutants. We speculate that Cas‐SF01's engineered amino acid substitutions enhance recognition specificity, mitigating short‐spacer risks, though future whole‐genome sequencing is warranted for comprehensive validation. Finally, editing efficiency was lower at the *ZmCCT* promoter (T3) than at the exon/UTR, likely because compact chromatin and high nucleosome occupancy restrict Cas‐SF01 accessibility. Nevertheless, the successful editing across diverse genomic contexts highlights the robust adaptability of our optimized Cas‐SF01 toolkit, paving the way for targeted improvement of varied agronomic traits in elite maize germplasm.

## CONFLICTS OF INTEREST

The authors declare no conflicts of interest.

## AUTHOR CONTRIBUTIONS

J.Z., R.W., and Y.L. conceived the study. M.L., Y.W., and L.Z. designed and performed the experiments. X.Z., Y.F., X.L., and Z.S. carried out genetic transformation. M.L. analyzed the data and wrote the manuscript. All authors have read and approved the contents of this paper.

## Supporting information

Additional Supporting Information may be found online in the supporting information tab for this article: http://onlinelibrary.wiley.com/doi/10.1111/jipb.70264/suppinfo

Figure S1. Percentages of deletion patterns produced by Strategies 1–5 for different targets
Figure S2. Plasmid schematic of the CRISPR/Cas‐SF01 gene‐editing toolbox for stable maize transformation
Figure S3. Generation and validation of *ZmCCT*‐edited transgene JING724 plants using the optimized Cas‐SF01 gene‐editing toolbox
Table S1. Information on targets used in this study
Table S2. Summary of genome‐editing efficiency and mutation profiles of single‐target Cas‐SF01 strategies at endogenous targets in the transgenic T_0_ maize inbred line JING724
Table S3. Summary of genome‐editing efficiency and mutation profiles of three targets connected in series using Cas‐SF01 strategies at endogenous targets in transgenic T_0_ maize inbred line JING724
Table S4. Sequence of elements used in this study
Table S5. List of oligonucleotides used in this study

## References

[jipb70264-bib-0001] Certo, M.T. , Gwiazda, K.S. , Kuhar, R. , Sather, B. , Curinga, G. , Mandt, T. , Brault, M. , Lambert, A.R. , Baxter, S.K. , Jacoby, K. , et al. (2012). Coupling endonucleases with DNA end‐processing enzymes to drive gene disruption. Nat. Methods 9: 973–975. 22941364 10.1038/nmeth.2177 PMC3602999

[jipb70264-bib-0002] Duan, Z. , Liang, Y. , Sun, J. , Zheng, H. , Lin, T. , Luo, P. , Wang, M. , Liu, R. , Chen, Y. , Guo, S. , et al. (2024). An engineered Cas12i nuclease that is an efficient genome editing tool in animals and plants. Innovation (Camb) 5: 100564. 38379787 10.1016/j.xinn.2024.100564 PMC10878114

[jipb70264-bib-0003] Hung, H.Y. , Shannon, L.M. , Tian, F. , Bradbury, P.J. , Chen, C. , Flint‐Garcia, S.A. , McMullen, M.D. , Ware, D. , Buckler, E.S. , Doebley, J.F. , et al. (2012). *ZmCCT* and the genetic basis of day‐length adaptation underlying the postdomestication spread of maize. Proc. Natl. Acad. Sci. U. S. A. 109: E1913–E1921. 22711828 10.1073/pnas.1203189109 PMC3396540

[jipb70264-bib-0004] Pan, W. , Gao, C. , Niu, D. , Cheng, J. , Zhang, J. , Yan, X. , Long, Q. , Zhu, Y. , Sun, W. , Xie, Q. , et al. (2025). Efficient gene disruption in polyploid genome by Cas9–Trex2 fusion protein. J. Integr. Plant Biol. 67: 7–10. 39451161 10.1111/jipb.13797

[jipb70264-bib-0005] Wang, W. , Li, S. , Yang, J. , Li, J. , Yan, L. , Zhang, C. , He, Y. , and Xia, L. (2025a). Exploiting the efficient Exo:Cas12i3‐5M fusions for robust single and multiplex gene editing in rice. J. Integr. Plant Biol. 67: 1246–1253. 39873911 10.1111/jipb.13850 PMC12060748

[jipb70264-bib-0006] Wang, W. , Yan, L. , Li, J. , Zhang, C. , He, Y. , Li, S. , and Xia, L. (2025b). Engineering a robust Cas12i3 variant‐mediated wheat genome editing system. Plant Biotechnol. J. 23: 860–873. 39690508 10.1111/pbi.14544 PMC11869199

[jipb70264-bib-0007] Wang, Y. , Zhang, L. , Liu, M. , Zhou, M. , Liu, X. , Song, Z. , Li, X. , Fu, Y. , Chen, H. , Liu, Y. , et al. (2026). Improved efficiency of genetic transformation for the development of elite germplasm using CRISPR/Cas12i3 in maize. aBIOTECH 7: 100017. 41940161 10.1016/j.abiote.2025.100017 PMC12973389

[jipb70264-bib-0008] Yang, Q. , Li, Z. , Li, W. , Ku, L. , Wang, C. , Ye, J. , Li, K. , Yang, N. , Li, Y. , Zhong, T. , et al. (2013). *CACTA‐like* transposable element in *ZmCCT* attenuated photoperiod sensitivity and accelerated the postdomestication spread of maize. Proc. Natl. Acad. Sci. U. S. A. 110: 16969–16974. 24089449 10.1073/pnas.1310949110 PMC3801022

[jipb70264-bib-0009] Zhang, H. , Li, Z. , Xiao, R. , and Chang, L. (2020). Mechanisms for target recognition and cleavage by the Cas12i RNA‐guided endonuclease. Nat. Struct. Mol. Biol. 27: 1069–1076. 32895556 10.1038/s41594-020-0499-0 PMC8256696

