# Supplementary material for: Optimized Cas‐SF01 gene‐editing toolbox shortens flowering timing in commercial maize inbred JING724
Source: J Integr Plant Biol. 2026 Apr 22;68(9):3178–80. doi: 10.1111/jipb.70264 (PMC13545821; doi:10.1111/jipb.70264)
Supplement: Supplementary file 1 [file JIPB-68-3178-s001.docx]

**Supplemental Data**

**Optimized Cas-SF01 gene-editing toolbox shortens flowering timing in commercial maize inbred JING724**

Mengyuan Liu^†^, Yao Wang^†^, Lu Zhang^†^, Xiang Zhang, Xiaqing Wang, Xinxiang Liu, Yue Fu, Xiaoxu Li, Ziheng Song, Ya Liu*, Ronghuan Wang*, Jiuran Zhao*

Beijing Key Laboratory of Maize DNA Fingerprinting and Molecular Breeding, Maize Research Institute, Beijing Academy of Agriculture and Forestry Sciences, Beijing 100097, China

^†^ These authors contributed equally to this work.

***Correspondences:** Jiuran Zhao ([maizezhao@126.com](mailto:maizezhao@126.com), Dr. Zhao is fully responsible for the distribution of all materials associated with this article); Ya Liu: ([liuya@maizedna.org](mailto:liuya@maizedna.org)); Ronghuan Wang (ronghuanwang@126.com)

# **Abbreviation of Figure 1A**

ZmU6-2, *Zea mays* U6 *Pol III* promoter variant 2;

crRNA, CRISPR RNA;

HDV, hepatitis delta virus ribozyme;

HH, hammerhead ribozyme;

tRNA-13, *Zea mays* glycine-tRNA variant 13;

Nos, terminator (*Oryza sativa T28* terminator is the first NOS and *Cauliflower Mosaic Virus 35S* terminator variant 01 is the second NOS);

prZmUbi, promoter of *Zea mays Ubiquitin-1* gene;

4*NLS, four nuclear localization sequences connected in series;

TREX2, Three Prime Repair Exonuclease 2.

# **SUPPORTING INFORMATION**

**Figure S1. Percentages of deletion patterns produced by Strategies 1****–5 for different targets.**

Error bars represent the means ± *SD*. Each dot represents a biological replicate.

**Figure** **S2.** **Plasmid schematic of the CRISPR/Cas-SF01 gene-editing toolbox for stable maize transformation.**

The pre-validated Strategy 4 construct was cloned into a vector carrying the PMI selection cassette and an embryogenesis-promoting cassette harboring with dual *WUS* and *BBM* genes at the *AscI* site. The recombinant vector was delivered into immature embryos of maize inbred line JING724 by *Agrobacterium* *tumefaciens*-mediated infection, and T₀ plants positive for the cassettes were obtained for detailed characterization of mutation profiles at the T1**–**T4 targets loci.

**Figure S3. Generation and validation of *ZmCCT*-edited transgene JING724 plants using the optimized Cas-SF01 gene editing toolbox.**

**(A)** Schematic of the three target sites in *ZmCCT* (exon, 5’-UTR and promoter). **(B-D)** PCR-based detection of the Cas-SF01 transgene and *ZmCCT* target locus in transgenic maize generations. PCR amplification of the Cas-SF01 expression cassette in T₀ transgenic plants (B) and T_3_ generation transgenic plants (C) confirming the Cas-SF01-free plants. PCR amplification of the *ZmCCT* target locus in T₃ generation plants, to verify the presence of the target gene locus in these Cas-SF01-free homozygous mutants before Sanger sequencing. The positive controls (+) were the transformation plasmid in B, C and the wild-type DNA in D, which showed clear bands, while the negative controls (**−**) were the wild-type DNA in B, C and ddH_2_O in D, which showed no amplification; lanes #1**–**#6 represent independent T₀ mutant lines in B and T_3_ mutant lines in C, D. M denotes the DNA marker where the brightest upper band corresponds to 1500 bp and the brightest lower band corresponds to 250 bp. **(E-G)** Representative Sanger sequencing chromatograms of T1 (E), T2 (F) and T3 (G) generated mutations. Green arrows indicate the crRNA, and the PAM is marked in red font. **(H)** Relative RNA expression of *ZmCCT* detected in the six types of mutations. *ZmActin* as control. Error bars represent the mean ± *SD* across five independent biological replicates. **(I)** Tassel branch number of the six types of mutations. Error bars represent the mean ± *SD* of 10 independent biological replicates. **(J)** Ear phenotypes of *Zmcct* #1, #3 and #5 lines. Bar= 10 cm. In H and I, statistical significance was evaluated by one-way ANOVA. Each dot represents a biological replicate. Different letters indicate significant differences at *P* < 0.05

**Table S1. Information on targets used in this study.**

**Table S2.** **Summary of genome editing efficiency and mutation profiles of single targets Cas-SF01 strategies at endogenous targets in transgenic T_0_ maize inbred line JING724.**

**Table S3 Summary of genome editing efficiency and mutation profiles of three targets connected in series of using Cas-SF01 strategies at endogenous targets in transgenic T_0_ maize inbred line JING724.**

**Table S4 Sequence of elements used in this study.**

**Table S5. List of oligonucleotides used in this study.**

# **MATERIALS AND METHODS**

**Construction of the vectors**

For maize protoplast experiments, the vectors were constructed based on the backbone of pUC-57 Kan. The DNA sequence of ZmU6-2-crRNA-HDV-termination sequence and the *Cas-SF01* gene were provided by Jinsheng Lai (China Agricultural University, China) and Jiankang Zhu (Southern University of Science and Technology, China) (Duan et al., 2024). Then *ZmU6-2-crRNA-HDV-termination*, *ZmU6-2-HH-crRNA-HDV-termination* and *ZmU6-2-tRNA-13-crRNA-HDV-termination* were amplified and inserted before the *ZmUBI-01* promoter of plasmid. *4*NLS-Cas-SF01-4*NLS-termination*, *4*NLS-CasSF01-Linker-TreX2-4*NLS-termination* gene, and *4*NLS-TreX2-Linker-CasSF01-4*NLS-termination* gene were amplified and inserted after *ubi* promoter of plasmid respectively. The construction of the above-mentioned plasmid facilitated by the NEBuilder^®^ HiFi DNA Assembly (New England Biolabs). Targets were designed by CRISPR-GE (<http://skl.scau.edu.cn/>) and were constructed with Golden Gate reactions. Table S1 provides a comprehensive list of the crRNAs utilized in this study. All sequence of element and primer pairs using in this study were listed in Table S2 and S3 respectively.

For maize stable transformation, Strategy 4 was constructed in *pCAMBIA1300* which including *PMI* gene as selectable marker driven by *CaMV35S* promoter, using *AscI* restriction sites. Meanwhile *BABY BOOM* (*Bbm*) and *WUSCHEL2* (*Wus2*) pattern (Mookkan et al., 2017) were fused after Cas-SF01 patter in order to improve transformation efficiency (Figure S1).

**Maize protoplast isolation and transfection**

Maize protoplast isolation and transfection were described (Xu et al., 2022). The JING724 was used in this study. Seeds were kept in the dark at 25℃ for 7**–**14 days until the second leaf was fully etiolated. The middle part (6**–**8 cm) of the second leaf was removed. Twenty leaves were placed in a pile (and pressed gently) and 0.5 mm strips were cut without wounding the leaves. The leaf strips were submerged in enzyme solution (1.5% cellulase R10, 0.4% macerozyme R10, 0.5 M mannitol, 20 mM KCl, 20 mM MES, pH 5.7, 10 mM CaCl_2_, 0.1% BSA) for another 6 h in dark with gentle shaking (60 r.p.m. on a platform shaker) until the solution turned yellow. Protoplast samples were viewed under a microscope to check for the release of round protoplasts. An equal volume of W5 solution (154 mM NaCl, 125 mM CaCl_2_, 5 mM KCl, 2 mM MES, pH 5.7) was added to the enzyme solution and the mixture was filtered using a 35**–**75 μm nylon mesh. The filtered solution was placed in a round-bottomed tube and spun at 100g and 4°C for 3 min to pellet the protoplasts. Protoplasts were resuspended in W5 solution on ice for 30 min, then spun at 100g and 4°C. The protoplasts were resuspended in MMG (0.4 M mannitol, 15 mM MgCl_2_, 4 mM MES, pH 5.7) solution. The subsequent transfection experiment was carried out at room temperature. Ten microlitres of DNA (10**–**20 μg) and 200 μl of protoplasts were added to a microfuge tube and mixed well. An equal volume of 40% (v/v) polyethylene glycol 4000 solution (4 g of polyethylene glycol 4000, 3 ml of H_2_O, 2.5 ml of 0.8 M mannitol, and 1 ml of 1 M CaCl_2_) was added, mixed well and incubated in the dark for 20 min. One millilitre of W5 solution was added to end the reaction and the mixture was spun at 100g for 1 min. The supernatant was removed and the protoplasts gently resuspended in 500 μl of W5 solution. Then the transfected protoplasts were incubated separately at 22 °C and 28 °C for 48 h. After incubation, genomic DNA was extracted with the Plant Genomic DNA Kit (Tiangen Biotech) used for NGS and the editing efficiency of Cas‑SF01 was compared under these two temperature conditions

***Agrobacterium tumefaciens*-mediated maize stable transformation**

The maize cultivar JING724 was used in this study. All binary vectors were introduced into *Agrobacterium* tumefaciens strain *LBA4404* using a freeze**-**thaw method. Maize embryogenic calli were then infected by *Agrobacterium*. We established a stable and efficient genetic transformation system for JING724 using the morphogenic genes *Baby boom* (*Bbm*) and *Wuschel* (*Wus2*) and the selection marker *6-phosphomannose isomerase* (*PMI*) to get stable transgene maize lines (Wang et al., 2026). Immature embryos (1.0**–**1.5 mm) from *Zea mays* inbred line JING724 at 10**–**12 days after pollination (DAP) were surface-sterilized with 50% (v/v) sodium hypochlorite, infected with *Agrobacterium tumefaciens* strain LBA4404 harboring a binary vector (Figure S2, containing *pmi* selection marker and *Bbm/Wus2* morphogenic genes), co-cultured in the dark for 2 days, recovered for 7 days, selected on medium with 1.2 g/L mannose under 24-h mixed light (6500K + 3000K LEDs) for ~9 days, regenerated on medium supplemented with 0.2 mg/L NAA for 20 days, and rooted for 21 days; positive T₀ transformants were verified by PCR amplification of the Cas-SF01 gene used prZmUbi1-01-Cas-SF01-F/R primer (Figure S3B and Table S5).

**Amplicon deep sequencing and data analysis**

Next generation sequencing (NGS) technology is applied in the analysis of PCR amplicons to detect and quantify gene editing efficiencies at specific target sites as described previously (Liu et al., 2024). The target region was amplified from all DNA with primers in the first round of PCR (Table S5). In the next round of PCR, both forward and reverse barcodes were added to the ends of the PCR products using barcode primers. The PCR products were sent for NGS (Tsingke Biological Technology, Beijing, China) using the MiSeq platform. Analysis of gene editing efficiency in maize protoplast experiment was performed using CRISPResso2 (Clement et al., 2019) to call indels. Mutation reads that were present in fewer than 1% of total reads were filtered during data analysis. Percentage of total transgenic plants was calculated as number of plants with mutation at target sites divided by the number of NGS samples. Percentage of correct edit plants was calculated as the number of homozygous, heterozygous, or chimeric mutations. The sequencing depth of each protoplast sample was about 1000× and of each transformation plant was about 200x.

**RNA extraction and qRT-PCR**

RNA extraction and reverse transcription quantitative polymerase chain reaction analysis were conducted as described previously (Yue et al., 2025) and 1 mg of total RNA was used for reverse transcription with the HiScript III 1^st^ Strand cDNA Synthesis Kit (Vazyme, China). RT-qPCR was conducted using the ChamQ Universal SYBR qPCR Master Mix (Vazyme, China) following the manufacturer's instructions. *ZmActin* was used as an internal control and the relative levels of gene expression were calculated using the 2^−ΔΔCt^ method. Each experiment was performed in biological triplicate, and a group of 5 seedlings was used as one biological replicate. All primers used in this study are listed in Supplementary Table 1.

**Data analysis**

Statistical significance among treatments was determined by Student's *t*-test or one-way ANOVA followed by Tukey's post hoc test using GraphPad Prism 9.5.0 software and Excel (2021).

# **REFERENCE**

**Clement, K., Rees, H., Canver, M. C., Gehrke, J. M., Farouni, R., Hsu, J. Y., Cole, M. A., Liu, D. R., Joung, J. K., Bauer, D. E., and Pinello, L.** (2019). CRISPResso2 provides accurate and rapid genome editing sequence analysis. Nat. Biotechnol. **37:** 224-226.

**Duan, Z., Liang, Y., Sun, J., Zheng, H., Lin, T., Luo, P., Wang, M., Liu, R., Chen, Y., Guo, S., Jia, N., Xie, H., Zhou, M., Xia, M., Zhao, K., Wang, S., Liu, N., Jia, Y., Si, W., Chen, Q., Hong, Y., Tian, R., and Zhu, J. K.** (2024). An engineered Cas12i nuclease that is an efficient genome editing tool in animals and plants. Innovation (Camb) **5:** 100564.

**Liu, M., Zhang, X., Xu, W., Kang, G., Liu, Y., Liu, X., Ren, W., Zhao, J., and Yang, J.** (2024). Efficient and precise genomic deletion in rice using enhanced prime editing. aBIOTECH **5:** 214-218.

**Mookkan, M., Nelson-Vasilchik, K., Hague, J., Zhang, Z. J., and Kausch, A. P.** (2017). Selectable marker independent transformation of recalcitrant maize inbred B73 and sorghum P898012 mediated by morphogenic regulators *BABY BOOM* and *WUSCHEL2.* Plant Cell Rep. **36:** 1477-1491.

**Wang, Y., Zhang, L., Liu, M., Zhou, M., Liu, X., Song, Z., Li, X., Fu, Y., Chen, H., Liu, Y., Wang, R., and Zhao, J.** (2026). Improved efficiency of genetic transformation for the development of elite germplasm using CRISPR/Cas12i3 in maize. aBIOTECH **7**.

**Xu, W., Yang, Y., Yang, B., Krueger, C. J., Xiao, Q., Zhao, S., Zhang, L., Kang, G., Wang, F., Yi, H., Ren, W., Li, L., He, X., Zhang, C., Zhang, B., Zhao, J., and Yang, J.** (2022). A design optimized prime editor with expanded scope and capability in plants. Nat. Plants **8:** 45-52.

**Yue, L., Liu, M., Liao, J., Zhang, K., Wu, W. H., and Wang, Y.** (2025). CPK28-mediated phosphorylation enhances nitrate transport activity of NRT2.1 during nitrogen deprivation. New Phytol. **245:** 249-262.
